# Supplementary material for: Effects of integrated hospital treatment on the default mode, salience, and frontal-parietal networks in anorexia nervosa: A longitudinal resting-state functional magnetic resonance imaging study
Source: PLoS One. 2023 May 30;18(5):e0283318. doi: 10.1371/journal.pone.0283318 (PMC10228763; doi:10.1371/journal.pone.0283318)
Supplement: S1 Table — (PDF) [file pone.0283318.s001.pdf]

S1 Table. An example of behavioral limitation (for a target body weight of 40 kg)

BW (kg)	Behavior	Communication	Bathing	Others
33	Restricted to the room		Wiping with a towel	
34				Permission to read books
35	Restricted to the ward		Showering once each week	
36		Permission to send letters	Showering twice each week	
37		Permission to receive letters		Permission to listen to music
38	Free movement within the hospital	Permission to make telephone calls	Showering three times each week	
39		Permission to receive telephone calls		Permission to perform handicrafts
40	Free movement within the hospital grounds	Unlimited visits	Bathing	

The table was reproduced from an article by Amemiya & Takii et al. (2011).

BW: body weight.
