# Supplementary material for: Effects of integrated hospital treatment on the default mode, salience, and frontal-parietal networks in anorexia nervosa: A longitudinal resting-state functional magnetic resonance imaging study
Source: PLoS One. 2023 May 30;18(5):e0283318. doi: 10.1371/journal.pone.0283318 (PMC10228763; doi:10.1371/journal.pone.0283318)
Supplement: S2 Table — (PDF) [file pone.0283318.s002.pdf]

S2 Table. Details of medication use

Medication use, no.	Patients with anorexia nervosa, n = 18		Healthy controls, n = 18
	Pre	Post	
Antidepressant	2	2	0
Antipsychotic	3	5	0
Antianxiety	1	0	0
Antidepressant and antipsychotic	1	0	0
Antidepressant and antianxiety	1	0	0
Antipsychotic and antianxiety	3	2	0
Antipsychotic and mood stabilizer	0	1	0
Antidepressant, antipsychotic, and antianxiety	1	1	0
Antidepressant, antipsychotic, and mood stabilizer	0	1	0
Total	12	12	0

The following medicines were used:

Antidepressant: fluvoxamine, mirtazapine, trazodone, and sulpiride

Antipsychotic: olanzapine, risperidone, aripiprazole, chlorpromazine, and levomepromazine

Antianxiety: quazepam, diazepam, ethyl loflazepate, and etizolam

Mood stabilizer: lamotrigine
