# Supplementary material for: Effects of integrated hospital treatment on the default mode, salience, and frontal-parietal networks in anorexia nervosa: A longitudinal resting-state functional magnetic resonance imaging study
Source: PLoS One. 2023 May 30;18(5):e0283318. doi: 10.1371/journal.pone.0283318 (PMC10228763; doi:10.1371/journal.pone.0283318)
Supplement: S1 Fig — (PDF) [file pone.0283318.s003.pdf]

S1 Fig. Resting-state network FC maps of interest in each group

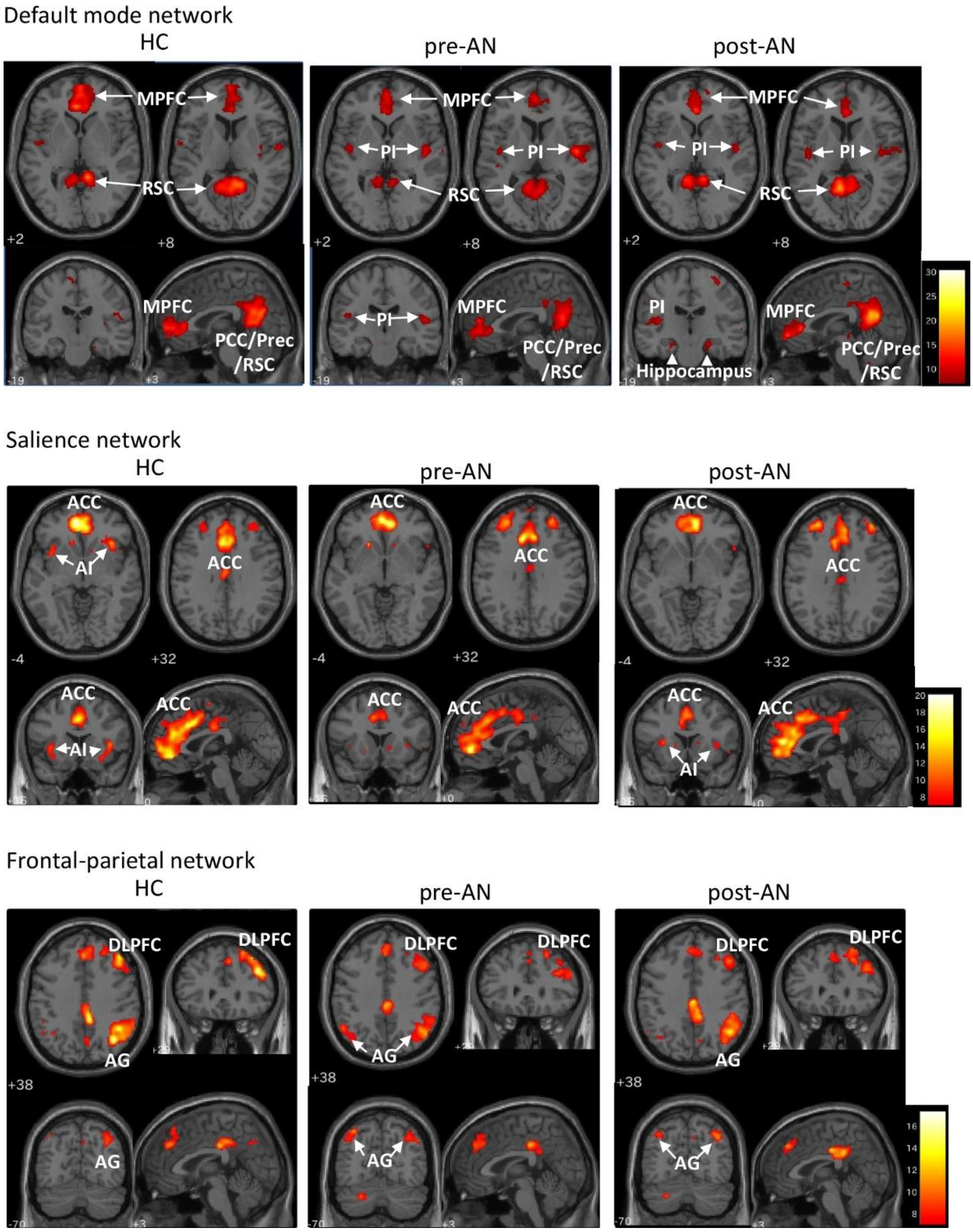

Spatial maps are plotted as *t* statistics thresholded at $p < 0.05$ and are family-wise error-corrected.

MPFC: medial prefrontal cortex, PI: posterior insula, RSC: retrosplenial cortex, PCC: posterior cingulate cortex, Prec: precuneus, ACC: anterior cingulate cortex, AI: anterior insula, DLPFC: dorsolateral prefrontal cortex, AG: angular gyrus.
