# Supplementary material for: Effects of integrated hospital treatment on the default mode, salience, and frontal-parietal networks in anorexia nervosa: A longitudinal resting-state functional magnetic resonance imaging study
Source: PLoS One. 2023 May 30;18(5):e0283318. doi: 10.1371/journal.pone.0283318 (PMC10228763; doi:10.1371/journal.pone.0283318)
Supplement: Attachment — Submitted filename: Response to Reviewers.docx [file pone.0283318.s004.docx]

The followings are the point-by-point responses to the questions and comments that were delivered in the letter dated November first.

The number of pages and lines are for the file " Revised Manuscript with Track Changes".

To both the reviewers: Thank you for giving us the opportunity to strengthen our manuscript with your valuable comments and queries. We incorporated your feedbacks in our manuscript and believe that these revisions improved it significantly.

Reviewer #1:

This study investigated abnormalities of the resting-state functional connectivity in the default mode network, salience, and frontal-parietal networks in the patients with anorexia nervosa. The functional connectivity in these three networks were evaluated cross-sectionally and longitudinally. This is very interesting study, there are however some issues to be mentioned.

1) The authors conducted many analyses and obtained many results. However, the manuscript seems to be redundant.

RESPONSE: The following tables and figures were moved to Supporting information.
S1 Table. An example of behavioral limitation

S2 Table. Details of medication use

S1 Fig. Resting-state network FC maps of interest in each group

- We removed some wording that appears to be repetitive. (p.4, lines64-66) (p.5, line82) (p.5, lines95) (p.11, lines225) (p.21, lines440-441) (p. 34, lines 753-755) (p. 34, lines 757)

2) In the Ethics section, the authors wrote ‘The current study was approved by the human research ethics committee at our hospital.’ What was ‘our hospital’?
   - RESPONSE: Corrected to “Kyushu university hospital.” (p.9, lines174)
3) In addition, the authors wrote ‘Written informed consent was obtained from all participants.’ Was this informed consent for the neuroimaging studies or/and cognitive behavioral approach with behavioral limitation?
   - RESPONSE: The informed consent is for both the neuroimaging studies and cognitive behavioral approach with behavioral limitation (p.9, lines 174-175).
4) In the Participants section, the authors wrote ‘All AN and HC participants were right-handed and aged between 15 and 50 years old.’ However, there was no description about parental consent for the adolescent participants in the Ethics section. This is a critical issue.
   - RESPONSE: Indeed, we obtained the consent from parents or guardians for minors, which is now added in the manuscript (p.9, lines 175-176).
5) ‘patients with severe depression, suicidal risk, personality disorders, schizophrenia, or alcohol dependence were excluded’ in this study. How about the obsessive-compulsive disorder that often comorbid with anorexia nervosa?
   - RESPONSE: We included the patients with mild or moderate depression, anxiety, or obsessive-compulsive symptoms, all of which are often comorbid with AN, which is now clearly stated in the manuscript.

(p.9, lines195)

“Patients with mild or moderate depression, anxiety, or obsessive-compulsive symptoms were included, because these symptoms are often comorbid with AN.”

6) How many numbers of male and female participants are there in each of the AN and HC group?
   - RESPONSE: All participants were female, which is now clearly stated (p.9, lines 181)
7) The author should write age range in both the AN and HC groups in the Table2.
   - RESPONSE: Corrected as suggested in the Table1. (p.21, lines 447)
8) Information of benzodiazepine should be written in the Table2.
   - RESPONSE: The use of benzodiazepines, in other words, antianxiety medications, is listed in S2 Table. After correcting the covariates and reanalyzing the relationship between RSN ROIs and psychological measurements, the correlation between TAS-20 DDF and FPN FC in AG was elucidated. RESULTS and DISCUSSION were rewritten accordingly (p. 27, lines 608) (p. 29, lines 652).
9) I could not understand the meaning of ‘AN-specific changes’ that the authors used. What the ‘AN-specific’ mean in this manuscript?
   - RESPONSE: We use 'AN-specific changes' as ‘changes in AN compared to HC’, which should mean symptoms and pathologies relevant specifically to AN.
10) Even though the AN patients received treatments, their mean BMI was 16.0, still small, and the mean total score of the EDI was 45.8, still high. Severity of the AN was changed from severe to mild, but the patients were not recovered. This is one of limitation in this study.
    - RESPONSE: We have reflected the reviewer’s comment in Limitation

(p. 35, lines 787-788).

“Third, many patients were mildly relieved by treatment but had not fully recovered at the time of discharge.”

Reviewer #2:

Remarks to the authors:

In the present study, the authors measured resting-state functional magnetic resonance images (MRi) from 18 patients with anorexia nervosa (AN) and 18 healthy subjects before and after integrated hospital treatment to examine hypotheses that the resting-state brain networks might be altered in such patients, and that treatment might normalize neural functional connectivity. (nourishment and psychological therapy). They showed that AN of post intervention exhibited higher functional connectivity (FC) in default mode network (DMN) and also higher FC in salience network (SN). The authors concluded results might reflect trait neural pathology regarding abnormal somatic perception, difficulty in socio-emotional processing, and excessive cognitive control in patients with AN. The manuscript is well written, and proposes interesting results in general. What follows are critical concerns and some suggestions to improve the manuscript.

Major Concerns:

1. It is strongly required for the authors to describe adopted denoising methods of their raw imaging data including motion correction and its exclusion criterion, which is currently obligatory for research of resting state functional MRI.
   - RESPONSE: We have stated the exclusion criteria we adopted in our head motion correction and confirmed that there was no significant head motion.

(p. 14, lines 305-308)

“According to the exclusion criteria for head motion correction in the previous resting state fMRI analyses (ref Tang et al 2022. and Trojsi et al 2022.), translational motion parameters were verified to be less than 1 functional voxel. Rotation motion parameters were verified to be less than 2 degrees.”

2. It is not popular to define the posterior insula as a part of default mode network, although these misleading findings could be found in results of an exploratory analysis of brain functional networks. It is highly recommended for the authors to interpret results based on understanding shared with those who are expertized in MRI studies, and to adopt appropriate expression to avoid misunderstanding of readers.
   - RESPONSE: We agree with you. I added that the inclusion of PI in DMN is special in AN.

(p. 24, lines 530-532)

“The PI is not generally considered a major region of the DMN. However, in our study, especially in AN, clusters in the PI were detected as part of the DMN.”

(p. 25, lines 560-561)

“The pre-AN group showed significantly higher DMN FC than HCs in the PI (Table 2 and Fig 1D), although PI is generally not the main region of DMN.”

Minor Concerns

1. The study design of the current study cannot also distinguish influence of intervention from change of body weight, in the other words malnourished state. The authors should discuss the point in the limitation section.
   - RESPONSE: We have added “Psychotherapy, as well as changes in body weight and medication use, may have affected the change in FC. (p. 35, lines 786-787).
2. In the introduction part (page 5, line 104), the authors stated that the previous studies showed inconsistent results. However, it is understandable that remitted AN exhibits different state from current AN (ie. ‘State or trait’ issue).
   - RESPONSE: We should have used more comprehensive expression, such as only ‘different study results in different states at different disease stages’.

(p. 6, lines 112-113)

“In previous studies of AN, analyses of the DMN have reported inconsistent results, such as different study results in subjects with AN in different states at different disease stages”

3. The authors should refer reasons why they adopt random effect model and its details, possibly with some previous references.
   - RESPONSE: Random effect analysis here means mixed effect including between-subject variance, which is normally used in SPM analysis procedure to simply indicate “second-level” group analyses that account for subject-to-subject variance. It might be confusing to use random effect here so we rephrased it as “second-level” analysis.

(p. 16, lines 355)

“with random effect second-level group analyses using voxel-by-voxel two-sample t-tests (SPM-12)”

(p. 17, lines 365)

“in random effect second-level group analyses using voxel-by-voxel paired t-tests (SPM-12)”

4. If some “sleep scale” was measured after resting state functional MRI to confirm participants were awake, it should be referred in the method section.
   - RESPONSE: Although the fMRI was performed at the first procedure with instructions beforehand not to fall asleep, we did not use a sleep scale. That is now referred to in the Method section:

(p. 13, lines 276-278)

“Although fMRI was performed at the first procedure with instructions not to fall asleep before the scan, a sleep scale was not used.”

5. The authors should precisely explain what component contribution values are (page 15, line 315). Do they stand for the strengths of within-network connectivity, or those of network connectivity to whole brain?
   - RESPONSE: We added “These values represent the strengths of within-network connectivity.” (p. 18, lines 387-388)
6. The authors stated that comparison of SDS between pre- and post-intervention was performed among 12 AN participants, but in the section “Relationship of RSN FC in ROIs to psychometric measurements” of page 15, the number of participants is 15 there.

- RESPONSE: Although we got SDS data from 15 pre-AN and 14 post-AN patients among 18 AN patients, we could only get 12 ‘paired’ sets of SDS at both pre- and post-AN timepoints that allows us to do longitudinal paired *t* tests. We made this clearer in the manuscript.

(p. 12, lines 254-256)

“Paired-sample *t*-tests were used to analyze a longitudinal subset of patients who completed questionnaires at pre- and post- AN (“pre-AN *vs.* post-AN,” SDS, n = 12; EDI, n = 16; TAS-20, n = 16) “

7. As for AN-specific ROIs, it is preferable for authors to carefully describe why they used the sphere mask from the peak voxel, instead of the mask of the region detected in the previous analyses.

- RESPONSE: We have added the reason as following:

(p. 18, lines 381-385) “In some cases, regions detected by the comparison were assumed to be small and consisted almost exclusively of peaks. To show that the peaks were not false positives and to correct for this possibility, the regions surrounding the peaks, including the peaks, were defined as the ROI.”

8. Just out of my curiosity, it would be welcomed if the authors show correlation between FC and body weight change, if possible. If no correlation was detected in that, it could be indirectly assumed that the FC change between pre- and post- was related to some effect of intervention.

- RESPONSE: We have already analyzed the correlation between pre-post change in each FC and weight change and found no correlation for any of the FCs, which is now stated in the manuscript.

(p. 20, lines 419-421)

“Additionally, correlation between a change of RSN FC in the ROIs and increase in BMI by treatment was calculated.”

(p. 28 lines 626-627)

“A change of RSN FC in any ROI was not significantly correlated with an increase of BMI.”
