# Supplementary material for: Effects of integrated hospital treatment on the default mode, salience, and frontal-parietal networks in anorexia nervosa: A longitudinal resting-state functional magnetic resonance imaging study
Source: PLoS One. 2023 May 30;18(5):e0283318. doi: 10.1371/journal.pone.0283318 (PMC10228763; doi:10.1371/journal.pone.0283318)
Supplement: Attachment — Submitted filename: Response to Reviewers.docx [file pone.0283318.s005.docx]

The followings are the point-by-point responses to the questions and comments that were delivered in the letter dated January 19th.

Reviewer #2: Remarks to the authors:

The authors have precisely approached all my comments and questions, and the manuscript has been considerably improved. What follows are suggestion to improve the manuscript.

Concerns:

1. In page 19 line 308, it is not assumed that the posterior insula might be detected in the group comparison, if the mask of default mode network (DMN) were appropriately applied. Careful interpretation and handling of this point would be recommended.
7. Still concerns in handling of posterior insula as if a part of DMN. The related expression in the discussion section should be carefully amended.

RESPONSE to the questions 1) and 7): In this study, the mask for the DMN was created in a data-driven fashion based on the ICA of the data obtained from current sample, in which the mask was chosen from one of the components from ICA that fitted best to the DMN a priori template. The reason why we did not use the a priori DMN template directly was that areas that function as default mode are not always constant as one fixed structural set of regions but may normally vary depending on the occasion to some extent – especially the characteristics of sample. Also the network functions and interactions within the networks are not stable, but rather, exhibit substantial variability across time and context (Dixon, et al. Interactions between the default network and dorsal attention network vary across default subsystems, time, and cognitive states. NeuroImage. 2017; 147:632-49). Our approach, creating the DMN mask specific to our study sample in a data-driven fashion, is to increase the sensitivity to detect more relevant regions that “actually functioned” as the default mode network, and has been used widely in the past studies and assumed valid and appropriate.

Through such a ROI setting process, there may be cases where the regions that have not been considered are detected as in the aimed network but closely connected to that network are selected as a part of the data-driven mask, such as the posterior insula (PI) relevant to the DMN. Even though the PI had not been assumed as a part of the DMN, the fact that the PI was selected as a part of the DMN mask indicates that the PI functioned as default mode in our sample (particularly in AN patients) and should be considered as a ROI in the subsequent analyses specifically in our study because it may include important information relevant to the AN pathology.

Nevertheless, according to the reviewer’s suggestion to avoid confusing the readers, we amended the description about the PI in the discussion where the PI had been referred to as if a part of the DMN:

(Page 32, line 556-573)

The right PI showed higher FC to the DMN FC in pre-AN patients than in HC. These phenomenaThe interaction between the DMN and PI might be associated with abnormal somatic sensation in patients with AN. The PI would be associated with pain and somatosensory processes [67, 69]. The patients with chronic low back pain showed high FC between the DMN and PI [70], which is regarded as a diseased phenomenon. AN patients also present bodily complaints frequently, so their high FC in the PI to the DMN FC in the PI might indicate the psychological modification of somatosensory information [71], which relates to their mind-induced somatic symptoms.

2. In page 19 line 316, it should be clarified whether a repeated measures ANOVA was adopted (recommended).

RESPONSE: We used paired-sample *t*-tests for a longitudinal subset of patients who completed questionnaires and scanning at pre- and post-AN as already described in the Method section in the manuscript, if we did not use covariations in the estimation model. Paired sample *t*-test is statistically equivalent to repeated measures ANOVA to compare changes over time at two time points.

3. In page 20 line 332, the use of sphere-shaped ROI has the risk that regions with different brain functions can be included. The authors should consider to cite an appropriate reference that shows validity of the method in analyzing resting state functional MRI instead they use labels that include peak voxels.

RESPONSE: The reason why we adopted the sphere ROIs centering the peak voxel was that only selecting the peak voxel as the ROI has the risk to pick up noise signals, so that some smoothing method is needed to denoise the information (i.e., adding the spatial widening like changing from peak to sphere). It has also a risk to use areas according to structural labels because areas only according to structural labels are too broad in many cases and do not necessarily reflect functional entity of the peak voxel in the analysis of time series BOLD signals in rsfMRI. Sphere-shaped functional ROI setting is a popular, conventional and safe method used in functional analysis of the brain and have been frequently used in recent rsfMRI studies [Spets, et al. 2021] [Pahapill, et al. 2020] [Martial, et al. 2019] [Chiang, et al. 2018] [Chaddock-Heyman, et al. 2018]. We adopted 5mm-radius sphere, which is small enough to avoid including functionally irrelevant brain regions outside the detected peaks.

4. In page 20 line 335-337, were mean values of the spheres calculated in these analyses?

RESPONSE: Yes, we calculated the mean of component contribution values within the voxels in ROIs in the individual component images. We clarify this in the manuscript.

(Page 18, line 312-314)

Next, we calculated the mean of component contribution values within the voxels in ROIs in the individual component image for pre-AN patients, HC, and post-AN patients using Marsbar software [41].

5. In page 22 line 364, just asking for confirmation purpose, was multiple comparison correction performed for 5 ROIs?

RESPONSE: Yes, the data from the 5 ROIs were analyzed with false discovery rate correction for multiple comparisons (page 19 line 336-342).

6. In page 27, the authors mentioned that posterior insula was detected especially in AN. Does it mean that it was visually obvious? It is a bit difficult for readers to understand, and the author should explain its implication.

RESPONSE: Yes, it is visually obvious. We had moved three RSNs in three group (HC, pre-AN, and post-AN) to supporting information (S1 Fig) in the last revision due to the heavy amount of information. However, we have displayed the DMN for HC and pre-AN in Fig 1 for the reader's convenience in the current manuscript.

Lastly, we would like to correct an error in the last manuscript revision in response to a minor concern raised by the reviewer #2, about what component contribution values are: the strengths of within-network connectivity, or those of network connectivity to whole brain.

(Page 18, line 312-315)

Next, we calculated the mean of component contribution values within the voxels in ROIs in the individual component image for pre-AN patients, HC, and post-AN patients using Marsbar software [41]. These values represent the strengths of within-network connectivity network connectivity to whole brain.
